# Protein-Directed Nucleation and Stabilization of Ultrasmall Silver Nanoparticles Within BSA Hydrogels

**DOI:** 10.3390/gels12030231

**Published:** 2026-03-12

**Authors:** Carmen Salto-Giron, M. Carmen Gonzalez-Garcia, Mari C. Mañas-Torres, Modesto T. Lopez-Lopez, Luis Alvarez de Cienfuegos, Jose L. Hueso, Angel Orte, Emilio Garcia-Fernandez

**Affiliations:** 1 Nanoscopy-UGR Laboratory, Departamento de Fisicoquímica, Unidad de Excelencia de Química Aplicada a Biomedicina y Medioambiente, Facultad de Farmacia, University of Granada, C. U. Cartuja, 18071 Granada, Spain; carmensalto@ugr.es (C.S.-G.);; 2 Departamento de Química Orgánica, Facultad de Ciencias, University of Granada, C. U. Fuentenueva, Avda. Severo Ochoa s/n, 18071 Granada, Spain; mariadelcarmen.manas@polymat.eu (M.C.M.-T.); lac@ugr.es (L.A.d.C.); 3 Basque Center for Macromolecular Design and Engineering (POLYMAT), Department of Applied Chemistry, Faculty of Chemistry, University of the Basque Country UPV/EHU, Paseo Manuel Lardizabal 3, 20018 Donostia-San Sebastián, Spain; 4 Research Unit “Modeling Nature” (MNat), Departamento de Física Aplicada, Facultad de Ciencias, University of Granada, C. U. Fuentenueva, 18071 Granada, Spain; modesto@ugr.es; 5 Instituto de Investigación Biosanitaria Ibs.GRANADA, Avda. de Madrid 15, 18014 Granada, Spain; 6 Instituto de Nanociencia y Materiales de Aragon (INMA), CSIC-Universidad de Zaragoza, Campus Rio Ebro, Edificio I+D, C/Poeta Mariano Esquillor, s/n, 50018 Zaragoza, Spain; jlhueso@unizar.es; 7 Department of Chemical and Environmental Engineering, University of Zaragoza, C/María de Luna, 3, 50018 Zaragoza, Spain; 8 Networking Research Center on Bioengineering, Biomaterials and Nanomedicine (CIBER-BBN), 28029 Madrid, Spain; 9 Instituto de Investigación Sanitaria (IIS) de Aragón, Avenida San Juan Bosco, 13, 50009 Zaragoza, Spain; 10 Escuela Politécnica Superior, University of Zaragoza, Crta. de Cuarte s/n, 22071 Huesca, Spain

**Keywords:** silver nanoparticles, green synthesis, protein hydrogels, bovine serum albumin, gel–nanoparticle composites, biocompatible

## Abstract

Biocompatible nanocomposite hydrogels are emerging as versatile platforms in nanomedicine, particularly when natural proteins are used as both structural and chemical components. In this work, we report a green, simple, and rapid in situ synthesis of ultrasmall silver nanoparticles (uAgNPs) within a bovine serum albumin (BSA) hydrogel, in which albumin simultaneously acts as the reducing agent and three-dimensional scaffold. The confined reaction environment generated uniformly dispersed Ag nanostructures with diameters in the 4–40 nm range, as confirmed by DLS and TEM. High-resolution TEM revealed clear Face-Centered Cubic (FCC, 111) lattice fringes, demonstrating the crystalline nature of the embedded uAgNPs. Quantitative image analysis showed narrow size distributions and high circularities, consistent with cluster stabilization through protein–metal interactions. Rheological measurements further indicated that the incorporation of uAgNPs enhanced hydrogel stiffness and delayed yielding, reflecting a reinforcement effect mediated by the nanoparticles acting as additional cross-linking points. Moreover, when very small embedded uAgNPs are formed, the presence of emissive silver nanoclusters was found using fluorescence emission spectroscopy. Overall, our results show that BSA hydrogels provide an effective matrix for directing green uAgNP nucleation, ensuring high stability, controlled growth in less than 2 min, and improved mechanical properties. The resulting protein–nanoparticle composite constitutes a promising soft material for imaging, sensing, and other biomedical applications requiring stable, biocompatible nanoscale architectures.

## 1. Introduction

The transition from synthetic polymer scaffolds to natural biopolymer-based hydrogels has redefined the design of advanced biomaterials for tissue engineering and drug delivery. Among natural proteins, serum albumin, specifically Bovine Serum Albumin (BSA), stands out due to its exceptional biocompatibility, non-immunogenic nature, and unique role as a multi-functional carrier in human physiology. The formation of three-dimensional albumin hydrogel networks through physical or chemical cross-linking provides a porous, hydrophilic scaffold that mimics the native extracellular matrix [1]. Integrating nanomaterials into these matrices transforms them into nanocomposite systems where the inorganic phase serves not only as a bioactive agent but also as structural reinforcement [2]. They have found diverse applications in sensing and biomedicine [3,4]. In particular, gel composites including embedded metallic silver nanoparticles (AgNPs) have been particularly interesting in the field of antimicrobial activity and wound healing [5,6,7,8].

The current state of research in this field is dominated by the pursuit of “green” and “one-pot” synthesis protocols [9]. Traditional methodologies often rely on hazardous chemical reductants like sodium borohydride (NaBH_4_) [10,11,12,13], which necessitate intensive purification to avoid systemic toxicity. Alternative methods employed photosensitized reduction, which required the utilization of a benzoine moiety [14], or photoinduced reduction and mild sugars [15]. Recent advances toward green synthesis of AgNPs emphasize the use of mild, biocompatible reducing agents such as glucose [16,17], plant extracts [6,18], or small molecules like tryptophan (Trp) [19] and other amphiphilic amino acids [20]. These latter approaches have successfully resulted in the implementation of protein-mediated synthesis of AgNPs. Among the reported processes, albumin, such as bovine serum albumin (BSA), has been employed to facilitate the in situ reduction of silver ions and subsequent stabilization of the resulting AgNPs [21,22]. However, in this process, the biophysical mechanisms underlying the reduction remain a subject of diverging hypotheses. A prominent controversy in the literature involves the identification of primary reducing residues. The “Tyrosine Hypothesis” suggests that the phenolic groups of tyrosine residues, which become deprotonated to tyrosinate in alkaline media (pH > 10), are the primary electron donors [23,24]. Conversely, the “Tryptophan Hypothesis” posits that Trp residues, due to their lower redox potential and location within hydrophobic pockets [25], act as central nucleation sites [19,21,23].

Once the protocol to yield AgNPs is optimized, two approaches for achieving AgNP-gel composites have been explored. On the one hand, AgNPs can be prepared and purified in a first step and subsequently added during the gellification process. This approach has been reported in polysaccharide hydrogels [6] and photocrosslinked alginate [13], among others [7]. However, a more interesting approach involves the in situ preparation of AgNPs within the gel matrix, either during gellification or in pre-formed gels. This ensures a uniform distribution of AgNPs throughout the matrix while avoiding the structural degradation often associated with multi-step fabrication [26,27]. The in situ reduction of Ag^+^ to AgNPs has been reported in hydroxypropyl methylcellulose-hydroxyapatite hydrogels [5], chitosan and hydroxypropyl methylcellulose hydrogels [16], supramolecular gels with amphiphilic hydrogellators [20], supra-molecular hydrogels of Fmoc-Phe and Phe–Phe [15,17], and hydrazone-based hydrogels [28]. In any case, the presence of NPs within the gel matrix usually has an impact on the mechanical properties of the gel, increasing structural stiffness, increasing porosity, and altering swelling kinetics, which in turn may be beneficial for biomedical applications [5,6,7,8].

In this context, the primary objective of this work is to develop a novel, rapid, and straightforward protocol for the one-pot synthesis of AgNPs embedded within an albumin hydrogel, and to assess the resulting physicochemical and rheological properties. In our protocol, gellification of BSA while fostering the in situ formation of size-controlled AgNPs yields composite materials with interesting properties. Our optimized green methodology successfully yields stable ultrasmall AgNPs (uAgNPs) in less than 2 min, highlighting its potential as a safe and robust platform for diverse biomedical applications.

## 2. Results and Discussion

### 2.1. Synthesis of AgNPs Within the BSA Hydrogel

**Figure 1:**
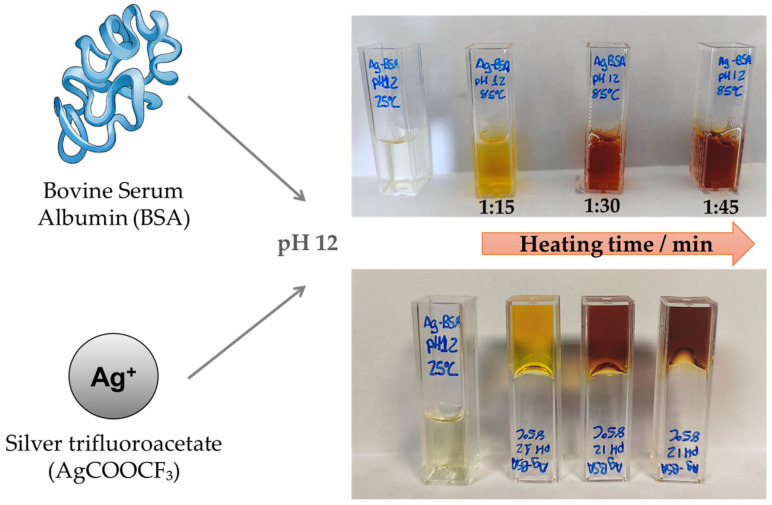
Visual evolution of uAgNP–BSA hydrogel composite formation, under alkaline conditions (pH 12), as a function of heating time (85 °C, min). Pictures of the resulting uAgNP–BSA hydrogel composites with gradual color change from pale yellow to brown as the heating time (min) increases are also shown.

Previous works reported the synthesis of AgNPs using BSA in an aqueous solution as a stabilizer [22]. However, this method yielded a broad size distribution of particles and exhibited certain limitations regarding reaction control and reproducibility. To address these issues, we expected that the in situ formation of AgNPs during the formation of thermally driven BSA hydrogels would show two-fold advantages. On the one hand, the formation of the gel would yield AgNPs of controllable sizes, and, on the other hand, result in composite materials. The experimental details of the synthesis method are described in Section 4; in brief, a BSA suspension containing AgCOOCF_3_ was heated up to 85 °C at pH 12 for a few minutes. During the synthesis process (Figure 1), the formation of Ag nanostructures and a BSA-hydrogel was macroscopically evident from the color, viscosity, and turbidity changes with time.

During thermal treatment under alkaline conditions, a progressive color change from pale yellow to brown was observed with increasing heating time. We obtained four samples of AgNP-BSA hydrogel composites at different heating times: 1 min 15 s (AgNPs 1:15), 1 min 30 s (AgNPs 1:30), 1 min 45 s (AgNPs 1:45), and 2 min (AgNPs 2:00). This chromatic evolution is characteristic of AgNP nucleation and growth and has been reported to correlate with progressive increases in nanoparticle size [29]. The heating temperature, 85 °C, was selected based on reports in the literature on both silver nanostructure synthesis and BSA gelation. Temperatures in the range of 70–90 °C are commonly employed for AgNP synthesis [22], while BSA gel formation requires temperatures exceeding its denaturation threshold (~62 °C), with optimal gelation reported near 85 °C [30]. When heating times exceeded approximately 5 min, irreversible BSA denaturation occurred, yielding a dark-brown, highly viscous liquid rather than a self-supporting gel. These observations define a very fast synthesis and a narrow processing window in which simultaneous gelation and controlled AgNP formation are achieved.

In our synthesis process, the concentrations of both BSA (50 mg/mL) and silver trifluoroacetate (10 mM) were higher than those employed previously in the liquid phase [22], resulting in several advantages. The elevated Ag(I) concentration enhanced AgNP yield, while the trifluoroacetate counterion, instead of the nitrate, contributed to the structural destabilization of BSA, promoting hydrogel formation and enhancing photostability of the AgNPs [22]. Simultaneously, increased BSA concentration and basic pH enhanced the reducing capacity of the system, enabling rapid Ag(I) reduction. Although we cannot rule out a certain contribution of Ag auto-reduction at this pH, the interaction with denatured BSA yields control during the reduction. In contrast to colloidal liquid systems, the gel matrix confines silver species spatially, limiting aggregation and favoring ultrasmall nanoparticle formation within the protein network.

### 2.2. Characterization of AgNPs

#### 2.2.1. Size of AgNPs

**Figure 2:**
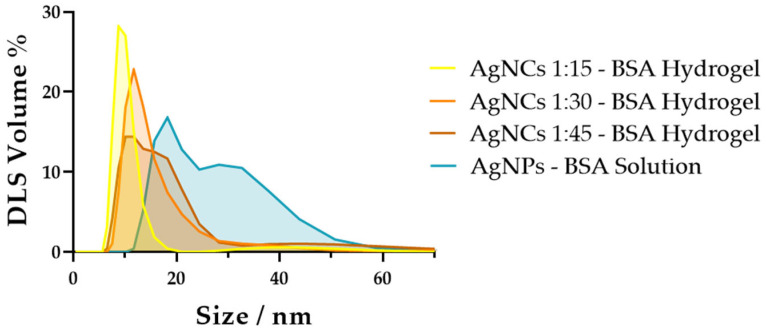
Volume-weighted particle size distribution of uAgNPs obtained in BSA hydrogels from three independent DLS measurements: AgNPs 1:15 (yellow), AgNPs 1:30 (orange), AgNPs 1:45 (brown), and AgNPs prepared with BSA in liquid phase following the protocol in [22] (blue).

To facilitate the characterization of the formed AgNPs using dynamic light scattering (DLS), we extracted the AgNPs from the BSA hydrogel by diffusion in water as described in Section 4.3. Then, we determined the hydrodynamic size and colloidal characteristics of the synthesized AgNPs (Table 1 and Figure 2). All measurements were conducted in deionized water, where AgNPs typically exhibit strong colloidal stability due to electrostatic repulsion and efficient surface capping. The volume-weighted distributions yielded a major population (>90% abundance) with a hydrodynamic diameter ranging from 9.6 to 14.6 nm, with an increase in size when comparing AgNPs 1:15 with AgNPs 1:30, and AgNPs 1:45. The corresponding average polydispersity index (PdI) of 0.57 ± 0.1 indicates a moderately polydisperse population, which is consistent with the appearance of a second minor population (abundance < 10%) with a larger diameter in the range of 50 nm. This apparent large polydispersity arises because scattering intensity scales with the sixth power of particle diameter (I ∝ d^6^). As a result, even a minor population of nanoparticle–protein complexes or transient aggregates disproportionately skews the measured size distribution toward larger particles. In any case, the more abundant population shows a narrow distribution supporting a homogeneous, size-controlled synthesis.

The hydrodynamic diameters obtained in this study are consistently lower than those typically reported for aqueous AgNPs synthesized using common stabilizers. AgNPs prepared by chemical reduction frequently exhibit hydrodynamic sizes ranging from ~25 to 120 nm, depending on core size, stabilizer concentration, and synthesis method. For example, AgNP synthesis by reducing with NaBH_4_ usually leads to diameters in the range of 20–70 nm [10,11,12]. Tejamaya et al. reported polyvinylpyrrolidone (PVP)-stabilized AgNPs with hydrodynamic diameters of 27–29 nm for particles with 10–12 nm metallic cores [31], while Ahlberg et al. described PVP-coated AgNPs with a metallic core of 70 nm and a hydrodynamic diameter of ~120 nm [32]. Similarly, Matea et al. demonstrated that citrate-, mercaptosuccinic acid-, and thioctic acid-capped AgNPs produced by wet chemical synthesis remained aqueously stable, with DLS confirming nanoscale hydrodynamic sizes characteristic of well-dispersed silver colloids [33]. In situ formation of AgNPs within supra-molecular hydrogels of Fmoc-Phe and Phe–Phe yielded AgNP sizes ranging from 60 to 160 nm [15,17]. Using biocompatible capping agents such as collagen and poly-L-Lysine yielded smaller particles (15 nm) through photoreduction but with broad distributions [14], indicating less controllable sizes.

Moreover, a direct comparison with BSA-stabilized AgNP synthesis methods reported in the literature showed diameters always larger than 20 nm [21,22]. For experimental comparative purposes, we also prepared AgNPs, utilizing BSA as a stabilizer in the liquid phase, adhering to the microwave-assisted procedure outlined by Basu and Mandal [22]. The AgNPs prepared using this protocol with BSA in solution (see Section 4.2) exhibited two populations of sizes in the range of 19–33 nm, with a much more abundant (>40%) large peak (Table 1 and Figure 2). In contrast, our synthesis approach produced substantially smaller AgNPs, demonstrating a higher degree of size control than what is commonly reported for water-dispersed colloidal silver systems. This reduction in particle size suggests that our method promotes more efficient nucleation and stabilization, enabling the formation of uniformly nanoscaled, ultrasmall nanoparticles (uAgNPs). Their controlled size distribution is particularly advantageous for applications requiring homogeneous nanoparticle–gel interactions, including antimicrobial formulations, catalytic gels, and nanocomposite materials. We used the term uAgNPs because their size, even though smaller than that of previous preparations, does not yet reach the typical size of Ag nanoclusters, nanoparticles composed of just a few metal atoms, usually measuring less than 2 nm, requiring a finely tuned templating agent [34,35,36].

Nevertheless, the fact that the uAgNP population that we measured was extracted from the gel by diffusion may bias the actual results of the particles formed within the gel. Indeed, very large particles may not readily diffuse out of the gel and ultrasmall AgNPs may remain linked to the gel by adsorption by physical interactions. Therefore, we further characterized the obtained AgNPs using high-resolution transmission electron microscopy (HR-TEM).

#### 2.2.2. HR-TEM Identification of Crystalline uAgNPs

**Figure 3:**
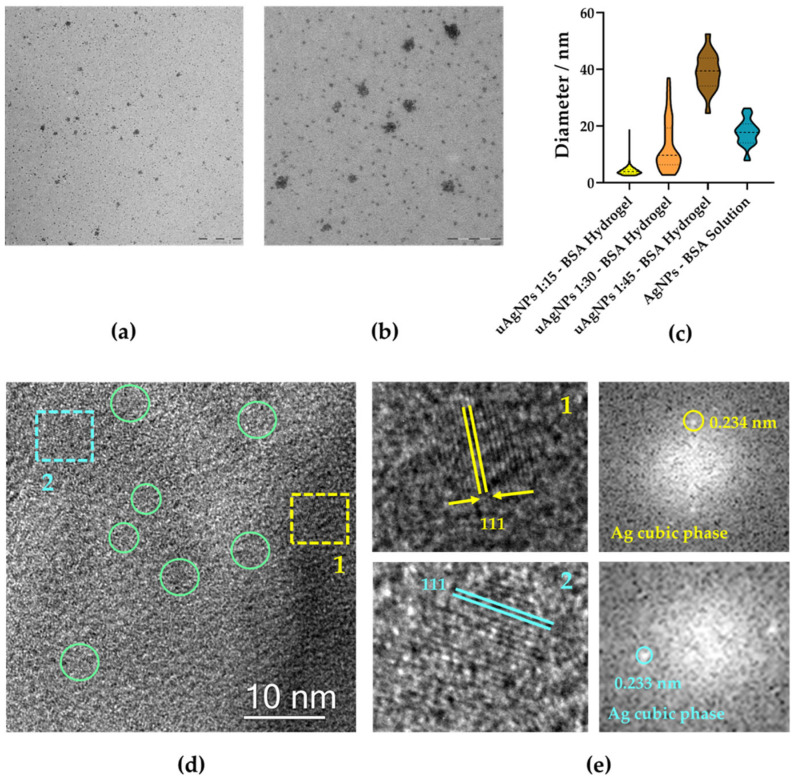
TEM and HR-TEM characterization of AgNPs embedded in a BSA hydrogel (heating time 1:15 min). (**a**,**b**) TEM images of AgNPs with (**a**) 500 nm and (**b**) 200 nm scale bars. (**c**) Violin plots of the diameter values obtained in the particle analysis of TEM images of AgNPs in uAgNP–BSA hydrogel composites from different reaction times, and AgNPs prepared with BSA in solution following the protocol in [22]. Error bars represent SD from three independent preparations. (**d**) A low-magnification image showing dispersed nanometric domains (green circles) embedded in the amorphous protein matrix. Dashed boxes (1 and 2) mark regions selected for high-resolution analysis. Scale bar: 10 nm. (**e**) Zoom 1 (yellow dashed box): Lattice fringes with a spacing of 0.234 nm, indexed to Ag(111); FFT confirms a single dominant periodicity. Zoom 2 (cyan dashed box): Lattice fringes with a spacing of 0.233 nm, likewise indexed to Ag(111) with matching FFT periodicity. The close agreement with the bulk Ag d(111) ≈ 0.235 nm supports assignment to crystalline FCC Ag nanoclusters stabilized within the BSA hydrogel network.

HR-TEM micrographs of the prepared AgNP-BSA hydrogel composites showed discrete nanoscale domains embedded in the amorphous, proteinaceous matrix, consistent with confined nucleation inside a crosslinked BSA environment (Figure 3 and Figure S1 in the Supplementary Materials, SM). The smooth, uniform background characteristic of the hydrogel contrasted with localized regions displaying well-defined lattice fringes, confirming the long-range atomic order typical of metallic Ag.

Quantitative image analysis provided size information. Particle geometry extracted from thresholded TEM images using an automated algorithm (see Section 4.4.3) revealed narrow size distributions and high isotropy in all the uAgNP–BSA hydrogel composites with different reaction times. uAgNP–BSA hydrogel composites heated for 1:15 min yielded a subpopulation of uAgNPs (Figure 3a,b), with a unimodal diameter centered at 4.2 ± 1.2 nm (Figure 3c), just in the limit of cluster-level confinement within the BSA matrix. Along the images, larger aggregates of particles were evident. However, closer inspection confirmed that those were formed by swarms of individual uAgNPs instead of coalesced aggregates. Extending the heating time to 1:30 and 1:45 min yielded larger structures (Figure 3c and Figure S1), yet both conditions maintained modest overall heterogeneity. The absence of a broad large-particle tail indicated that the hydrogel matrix effectively restricted coalescence and inhibited Ostwald ripening, thereby preserving ultrasmall particle sizes [37], especially at short incubation times. Circularity values remained consistently high, 0.87 ± 0.17 (AgNPs 1:15) and 0.89 ± 0.14 (AgNPs 1:30), with most particles falling within 0.85–1.0, confirming predominantly isotropic, near-spherical projected shapes (Figure S2). Minor deviations from perfect circularity likely reflected the expression of low-energy facets, predominantly (111), which was consistent with the lattice spacings identified by HR-TEM (see below). An additional observation from our TEM analyses was that uAgNPs formed in the BSA hydrogel heated for 1:45 min or longer undergo a distinct morphological transformation. Beyond this threshold, the ultrasmall confined AgNPs reorganized into larger, well-defined nanospheres with an average diameter of 39 ± 6.5 nm and circularity of 0.82 ± 0.09 (Figure 3c, Figures S1 and S2). This thermally induced transition suggested that elevated temperatures partially overcame the kinetic and steric stabilization imparted by the BSA matrix, allowing the system to minimize surface energy via particle coalescence or rearrangement into spherical particles.

The comparison of these size values with those obtained by DLS must be taken carefully. For instance, DLS measurements performed after diffusion-based extraction of the nanoparticles into water revealed larger hydrodynamic diameters for the uAgNPs 1:15 (approximately 8–10 nm), together with a relatively high polydispersity index (PdI ≈ 0.5). This result is expected, as DLS reports the hydrodynamic size of scattering objects in solution, which includes not only the metallic core but also the associated BSA corona [38,39], and the surrounding hydration layer. Therefore, TEM and DLS provide complementary information, with TEM resolving the metallic core dimensions and DLS reporting the larger hydrodynamic diameter of the nanoparticle–protein complexes dispersed in solution after extraction from the gel, which may also partially bias the results towards more mobile particles.

Notably, the morphology of AgNPs obtained by following the reference protocol in [22], using BSA in the sol state, showed a striking contrast. Under the less viscous conditions in this anisotropic synthesis, directional growth was facilitated, yielding a heterogeneous population of AgNPs and Ag nanorods averaging 17.6 ± 4.6 nm in size and subsequently lower circularity values of 0.65 ± 0.23 (Figure 3c, Figures S1 and S2). This difference highlights an important consequence of BSA hydrogel confined nucleation, which suppressed anisotropic particle evolution, yielding isotropic uAgNPs at short heating times and nanospheres later, but never nanorods, even when heated for longer times. This comparison underscores the strong morphological control exerted by the BSA matrix.

Finally, we employed HR-TEM to investigate the successful formation of crystalline silver nanostructures within the BSA hydrogel network. Representative regions (Figure 3d,e) exhibited interplanar spacings of 0.234 and 0.233 nm, matching the (111) planes of FCC silver (bulk d_111_ ≈ 0.235 nm) [40]. Corresponding Fast Fourier Transform (FFT) patterns showed a single dominant periodicity, providing unambiguous evidence that the embedded domains are crystalline uAgNPs.

#### 2.2.3. EDX Analysis and Elemental Composition

Energy-dispersive X-ray spectroscopy (EDX) was employed to verify the chemical composition of the uAgNPs observed by TEM (Figure S3). The spectrum unequivocally confirms the presence of elemental silver through multiple characteristic lines, including Ag Lα (~2.98 keV), Ag Lβ (~3.15 keV), and Ag Lγ/Ag M lines (~3.5–3.9 keV). Matrix-related elements (C, O, N, S) originate from the BSA peptide backbone and side chains. The detection of these elements supports the direct interaction of AgNPs with protein residues that can act as both soft templates and chemical stabilizers that restrict particle growth to the nanocluster regime. Previous studies have reported the interaction of AgNPs with BSA through amino, amide, and carboxyl groups [10], thiols of cysteine residues [41], hydroxyl groups of tyrosines [18], and the indole moieties of tryptophan (Trp) residues [18,19]. Our results confirm the role of such residues in the formation and stabilization of uAgNPs.

### 2.3. Rheological Characterization of the uAgNP–BSA Hydrogel Composites

**Figure 4:**
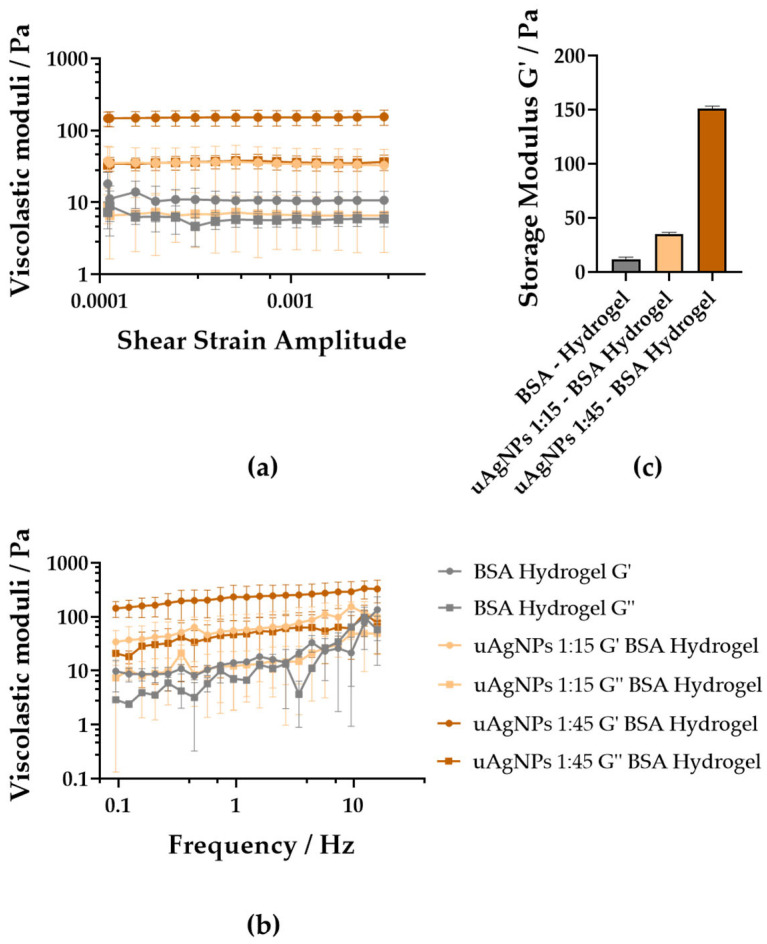
Rheological analysis of uAgNP–BSA hydrogel composites: BSA hydrogel (uAgNP-free, gray), uAgNPs 1:15 gel (light orange), and uAgNPs 1:45 gel (brown). (**a**) Amplitude sweep (0.01–0.2% strain, 1 Hz) illustrating the linear viscoelastic regime (LVR), showing G′ (circle) and G″ (square). (**b**) Frequency sweep (0.1–16 Hz, 0.1% strain) showing the storage modulus (G′) (circle) and loss modulus (G″) (square). (**c**) Comparison of G′ corresponding to the LVR at 1 Hz for different formulations.

The rheological response of the nanocomposite hydrogels aligned closely with the nanoscale organization revealed by DLS and TEM and was compared meaningfully with values reported for similar protein-based hydrogels. The confined, crystalline uAgNPs embedded within the BSA network acted as rigid nanoscale crosslinking points, restricting chain mobility and reinforcing the matrix. This was reflected in the frequency sweeps, where the Ag-containing gels (uAgNPs 1:15 and 1:45) displayed a dominant elastic response (G′ ≫ G″) and markedly higher G′ values than the nanoparticle-free controls (BSA hydrogel) (Figure 4). Comparable behavior has been reported for conventional BSA hydrogels, which exhibited solid-like viscoelastic profiles under oscillatory shear, although their moduli remain strongly dependent on pH and protein conformation [42]. Similarly, composite BSA-based hydrogels (e.g., BSA-polysaccharide systems) showed increased G′ upon incorporation of rigid network modifiers, demonstrating that embedded inclusions can densify the crosslinked network and enhance stiffness [43]. Here, the uAgNPs produce a comparable reinforcement effect, as evidenced by the extended linear viscoelastic regime and delayed yielding in amplitude sweeps. This monotonic increase in G′ mirrors classical nanocomposite toughening mechanisms: nanoscale fillers distribute strain, hinder network relaxation, and delay structural breakdown. Altogether, the rheology of the uAgNP–BSA hydrogels is consistent with, but significantly strengthened beyond, the baseline behavior of protein-only gels, confirming that the embedded silver nanostructures function as mechanically active nodes that measurably reinforce the hydrogel network.

To quantitatively rationalize this reinforcement, the network mesh size (*ξ*) of the BSA hydrogel was estimated from the plateau storage modulus (G′) using classical rubber elasticity theory [44]:

(1) $$\xi \approx {\left(\frac{k_{B}T}{G^{\prime }}\right)}^{1/3}$$

Using this approximation, the calculated mesh size was approximately 30 nm for the gel containing 4.2 nm uAgNPs (G′ = 155 Pa) and 23 nm for the gel containing 39.1 nm uAgNPs (G′ = 326 Pa). Comparison between nanoparticle radius (*R_NP_*) and network mesh size reveals two distinct structural regimes. For the uAgNP–BSA hydrogel composites prepared with 1:15 min of incubation (*R_NP_* ≈ 2.1 nm), the confinement parameter, *R_NP_*/*ξ* ≈ 0.07, indicated that the uAgNPs were substantially smaller than the characteristic pore size of the network. In this regime, the particles are likely accommodated within the hydrogel mesh with limited perturbation of network connectivity, resulting in moderate mechanical reinforcement (G′ = 155 Pa; tan *δ* = 0.316). In contrast, for the uAgNP-BSA hydrogel composites prepared with 1:45 min of incubation (*R_NP_* ≈ 19.5 nm), the quotient *R_NP_*/*ξ* yielded 0.85, placing the system in a geometrically coupled regime where the AgNP dimensions approach the mesh size. Under these conditions, AgNPs can no longer be considered passive inclusions but instead act as physical constraints within the network. This size matching promotes mechanical coupling between the protein strands and nanostructures, resulting in a more than two-fold increase in G′ (326 Pa) and a decrease in tan *δ* (0.233), indicating enhanced elastic character and suppressed network relaxation.

Importantly, the reinforcement does not scale with specific surface area (which would predict a stronger effect for smaller nanoparticles), but instead correlates with the dimensionless confinement parameter $R_{NP}/\xi$. This indicates that geometric compatibility between filler size and network architecture is the dominant factor governing viscoelastic enhancement in these uAgNP–BSA hydrogels [45,46].

Previous theoretical models by DFT supported the idea that silver atoms interact strongly with the amino and hydroxyl groups, creating stable Ag–N or Ag–O chemical bonds that physically anchor the metal to the protein scaffold [16]. To gain more insights into this interaction that has rheological impact on the gels, FTIR spectroscopy was employed to evaluate the interaction between BSA and silver species within the hydrogels (Figure S4). The BSA gel control showed the expected amide I (≈1640–1650 cm^−1^), amide II (≈1530–1540 cm^−1^), and several amide III–related peaks (1398, 1359, 1164, 1072 cm^−1^) bands, which were also present in the uAgNP–BSA hydrogel composites, although with slight broadening and minor intensity changes. These bands often shift in intensity when BSA interacts with silver nanoparticles due to the involvement of carboxylate, amide, and amino functional groups [10,38,47]. Our results show spectral shifts in the 1400–1450 cm^−1^ band, attributed to COO^−^ stretching, and the 1000–1200 cm^−1^ region, assigned to C–N and C–O vibrations (Figure S4). The uAgNP 1:45–BSA hydrogel composite exhibited a ~30 cm^−1^ shift near 1400 cm^−1^ and a ~100 cm^−1^ shift around 1200 cm^−1^ compared to the BSA hydrogel, indicating interaction between carboxylate and amide-related groups and the silver surface. This effect was less notable in the uAgNP 1:15–BSA hydrogel sample due to the smaller size of the uAgNPs (~4 nm), which can fit within the BSA network. The interaction with the AgNPs formed in the 1:45 min sample was stronger, correlating spectral shifts in FTIR with a larger rheological effect.

Overall, these findings demonstrate that Ag nanostructures function as mechanically active nodes whose reinforcing efficiency is maximized when their dimensions approach the intrinsic mesh size of the protein network.

### 2.4. Optical Characterization of the uAgNP–BSA Hydrogel Composites

We then investigated the optical properties of BSA-based hydrogels incorporating silver nanostructures and the influence of the morphology of the metallic component and its interaction with the protein matrix. One of the main features of AgNPs is their plasmonic nature. However, our uAgNP–BSA hydrogel composites showed the absence of a clearly defined localized surface plasmon resonance (LSPR) band in the visible region (Figure S5a). This was consistent with previous reports describing strong plasmon damping in ultrasmall silver nanoparticles and clusters [48,49]. At these sizes, collective electron oscillations were significantly suppressed due to a combination of increased surface scattering, quantum size effects, and strong coupling with the surrounding dielectric environment provided by the BSA hydrogel network [50]. In contrast, AgNPs and nanorods (20–30 nm) synthesized with aqueous BSA exhibited substantially higher absorbance across the UV–visible region. Although distinct transverse or longitudinal plasmon modes were not clearly resolved, the enhanced absorption could be attributed to the larger metallic volume of the nanorods and their anisotropic geometry, which increased the light–matter interaction even when classical plasmon bands were broadened or damped by protein adsorption and matrix confinement [50].

**Figure 5:**
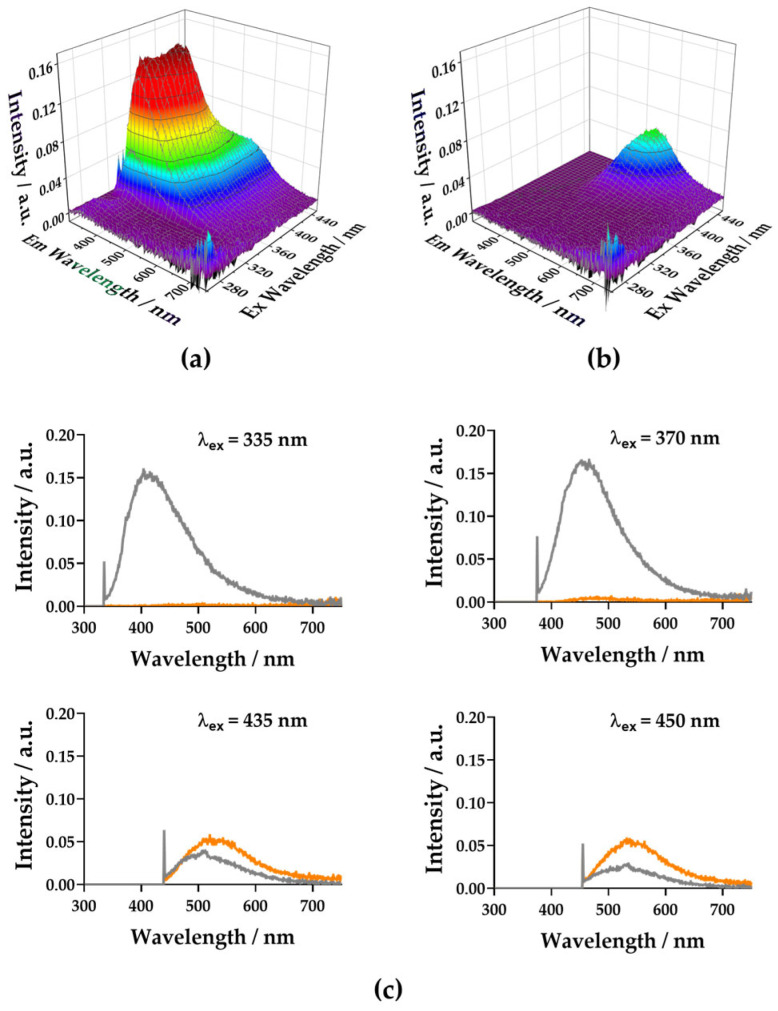
EEM matrixes of the BSA hydrogel 50 mg/mL (**a**), and uAgNPs 1:15–BSA hydrogel composite (**b**). (**c**) Emission spectra at different excitation wavelengths, extracted from the EEMs of the BSA hydrogel (gray) and uAgNPs (orange).

To test the presence of emissive Ag species embedded within the BSA hydrogel, we collected three-dimensional excitation–emission matrix (EEM) measurements (Figure 5a). The BSA hydrogel exhibited the characteristic tryptophan fluorescence signature of BSA, with excitation centered at ~275–280 nm and a relatively narrow emission band around 330–350 nm. This emission region corresponds to the dominant contribution of Trp residues and reflects the local polarity of the protein environment. Nevertheless, the BSA hydrogels also exhibited excitation-dependent, long-wavelength emission (Figure 5b). This emission may be attributed to long-range emissive interactions, usually detected in proteins and peptides [51]. In contrast, the uAgNP–BSA hydrogels displayed modified EEM surfaces. The most striking feature is the almost total disappearance of Trp emissions. Many previous reports studying the interaction between AgNPs and BSA reported readily static quenching of the BSA–tryptophan emission by the NPs [10,12,18,19]. In our case, the high concentration of uAgNPs within the gel resulted in total quenching. The EEM maps further showed the remaining excitation-dependent, long-wavelength emissions in the uAgNP–BSA hydrogels. Direct comparison of the spectral emission profiles at λ_ex_ in the 430–450 nm range (Figure 5b) demonstrates that the uAgNP–BSA hydrogels presented higher, red-shifted emissions than the BSA hydrogel alone. This indicates the presence of an additional emissive species beyond long-range protein fluorescence. This result suggests radiative contributions from Ag-related emissive centers stabilized within the protein matrix. These emissive species may be in the sub-2 nm regime, consistent with Ag nanoclusters. To confirm this, we obtained time-resolved fluorescence decays across the 550 nm band, with excitation at 450 nm (see Section 4.6 and Figure S6). The BSA hydrogel exhibited an average intensity-weighted lifetime of 1.88 ± 0.03 ns, consistent with partially quenched tryptophan fluorescence in a crosslinked protein network. In contrast, the uAgNP–BSA hydrogel composite exhibited an enhanced average lifetime of 3.75 ± 0.02 ns. If the observed emission was solely due to intrinsic Trp fluorescence, the presence of AgNPs would be expected to induce quenching via energy or electron transfer processes, typically shortening the lifetime. Instead, the observed lifetime extension indicates the contribution of a distinct emissive population. In fact, lifetime values in the 2–4 ns range are typical of biomolecular-templated Ag nanoclusters [22,36]. This supports the idea that the heterogeneous population of uAgNPs achieved within the BSA hydrogel contains a certain population of smaller, emissive silver centers.

## 3. Conclusions

This study presents a simple, biocompatible strategy for generating uAgNPs within a BSA hydrogel, enabled by the protein’s dual function as a reducing agent and confinement scaffold. The absence of additional strong reducing agents makes this synthesis biocompatible and green. TEM, HR-TEM, EDX, and DLS confirmed the formation of well-defined, crystalline uAgNPs whose size increased controllably with heating time, while remaining restricted by the hydrogel network. Rheological analysis demonstrated that in situ-formed uAgNPs strengthened the BSA matrix. The uAgNP–BSA hydrogel composites exhibited higher elastic moduli, an extended linear viscoelastic range, and delayed yielding compared to BSA-only controls, indicating that the confined uAgNPs act as reinforcing nanoscale crosslinkers. Moreover, fluorescence measurements showed the expected quenching of BSA fluorescence emissions driven by interactions with uAgNPs, but excitation at long wavelengths exhibited additional emissions that can be attributed to emissive Ag nanoclusters.

Our results highlight protein hydrogels as effective platforms for precise, matrix-guided nucleation and growth, stabilizing and tuning the properties of ultrasmall metal nanostructures. The resultant uAgNP–BSA hydrogel composites offer a promising soft material architecture for applications in fluorescence-based sensing, imaging, and biofunctional materials, including biomedical applications such as controlled release, bioimaging, wound healing, and antimicrobial patches.

## 4. Materials and Methods

### 4.1. Materials

Silver trifluoroacetate (AgCOOCF_3_), bovine serum albumin (BSA), and sodium hydroxide (NaOH) were used as received without further purification. All reagents were purchased from Sigma-Merck (Darmstadt, Germany). Ultrapure water (MilliQ grade) was used for all solution preparations and synthesis steps.

### 4.2. Synthesis of uAgNP–BSA Hydrogel Composites

The uAgNP–BSA hydrogel composites were synthesized by mixing AgCOOCF_3_ (10 mM) and BSA (50 mg/mL) in MilliQ aqueous solution, and the pH was adjusted to 12 by the addition of 1 M NaOH. The reaction mixture was heated in a thermostated water bath at 85 °C until the solution became viscous and developed an intense yellow color (1.25–2 min). The water-bath approach allowed continuous stirring and uniform thermal transfer, improving reproducibility and minimizing thermal degradation. The reaction was quenched by immersion in an ice bath, after which the samples were cooled to room temperature and stored at 4 °C until further use.

For comparison purposes, we also prepared AgNPs, using BSA as a stabilizer in the liquid phase, following the microwave-assisted procedure described by Basu and Mandal [22].

### 4.3. Purification of uAgNPs

The uAgNPs were extracted from the gel matrix to facilitate characterization by DLS. A portion of the hydrogel was manually fragmented to increase the surface area and transferred to a tube. MilliQ water was added to maintain a gel-to-water ratio of 1:10 (*w*/*v*) and stirred at room temperature, ensuring effective diffusion. Under these conditions, uAgNPs diffused out of the hydrogel to the aqueous phase separated using a pipette.

### 4.4. Characterization of uAgNPs

#### 4.4.1. Dynamic Light Scattering (DLS) Analysis

DLS analysis was performed to determine the hydrodynamic diameter and size distribution of the uAgNPs using a Malvern Zetasizer Micro V spectrometer (Malvern Instruments, Malvern, UK). Measurements were performed in triplicate at 25 °C with an equilibration time of 120 s before data acquisition. Samples were analyzed using the manufacturer’s software, applying the metallic nanoparticle model to obtain the volume-weighted diameter.

#### 4.4.2. Transmission Electron Microscopy (TEM) and Energy-Dispersive X-Ray Spectroscopy (EDX)

TEM samples were prepared by suspending the sample in water and dropcasting 5 μL of it onto a 200-mesh holey carbon grid (Electron Microscopy Sciences; Hatfield, PA, USA), followed by air-drying using anti-capillary tweezers. TEM measurements were performed using a LEO 906E transmission electron microscope from Carl Zeiss (Oberkochen, Germany), high-Resolution TEM was performed on a Thermo Fisher Scientific TITAN Imagen (Thermo Fisher Scientific; Waltham, MA, USA), and EDX spectroscopy was performed on a Thermo Fisher Scientific INSPECT-F50 SEM instrument. Additionally, TEM was also performed using a Tecnai T20 system (Thermo Fisher Scientific) operated at 200 kV, with a resolution of up to 0.24 nm. Aberration-corrected scanning transmission electron microscopy (Cs-corrected STEM) images were acquired using a high angle annular dark-field detector (HAADF) in a FEI XFEG TITAN electron microscope (FEI, Eindhoven, The Netherlands) operated at 200–300 kV. Elemental analysis was performed using an EDAX detector in scanning mode. Three uAgNP–BSA hydrogel composites were imaged, with uAgNPs obtained using heating times of 1 min 15 s, 1 min 30 s, or 1 min 45 s. For comparison purposes, AgNPs synthesized following the reference protocol [22] were used as a control. Part of the characterization of materials has been performed by the Platform of Production of Biomaterials and Nanoparticles of the NANBIOSIS ICTS, more specifically by the Nanoparticle Synthesis Unit (Unit 9) of the CIBER in Bioengineering, Biomaterials & Nanomedicine (CIBER-BBN).

#### 4.4.3. Morphometric Analysis of uAgNPs

TEM images were analyzed in FIJI (distribution of ImageJ v1.54p) [52] using a calibrated segmentation workflow. After contrast enhancement and light smoothing, particles were segmented by global MaxEntropy thresholding and quantified within a defined ROI to avoid thickness gradients. The minimum analyzed area was 5 nm^2^ (equivalent circular diameter ≈ 2.5 nm). Size metrics (area, Feret diameters) and circularity (4πA/P^2^) were computed on calibrated binary masks with edge particles excluded.

### 4.5. Rheological Measurements

The mechanical properties of the hydrogels were assessed under oscillatory shear using a rotational rheometer equipped with a 35 mm serrated double-plate geometry (P35 Ti L S, Thermo Fisher Scientific) to prevent wall slip. All measurements were performed at 37.0 ± 0.1 °C. Three hydrogel formulations were evaluated: 50 mg/mL BSA hydrogel (control), uAgNPs 1:15–BSA hydrogel, and uAgNPs 1:45–BSA hydrogel. For each formulation, amplitude sweep tests were first conducted at a fixed oscillation frequency of 1 Hz, while the shear strain amplitude (γ_0_) was increased logarithmically from 0.0001 to 0.002. These measurements provided the storage modulus (G′) and loss modulus (G″) as functions of strain and enabled the determination of the linear viscoelastic region. Subsequently, frequency sweep tests were performed at a constant shear strain amplitude of γ_0_ = 0.001, while the oscillation frequency was increased stepwise from 0.1 to 16 Hz. These measurements yielded the mechanical spectra of the gels, reporting G′ and G″ as functions of frequency. A fresh hydrogel sample was loaded for each amplitude sweep, frequency sweep, and experimental condition to avoid structural fatigue or measurement history effects. For every formulation, at least three independent samples were analyzed, and the mean values and standard errors are reported.

### 4.6. FTIR, UV–Vis Absorption and Fluorescence Emission Spectroscopy

FTIR measurements were performed using a FT/IR-4600 type A spectrometer (JASCO; Tokio, Japan). All spectra were collected using the ATR mode with a spectral resolution of 4 cm^−1^. The hydrogels were analyzed by cutting a small portion of each sample and placing it directly onto the ATR crystal to ensure full surface contact. Instrumental parameters included 60 accumulations, cosine apodization, automatic gain and aperture settings, and a data interval of 0.964 cm^−1^. The spectra of the BSA hydrogel and the uAgNP–BSA hydrogel composites were acquired under identical conditions.

To characterize the optical properties of the uAgNPs, three aqueous solutions were prepared under synthesis-equivalent conditions: BSA (50 mg·mL^−1^, pH 12, 85 °C 1:15 min, diluted 1:20), AgCOOCF_3_ (10 mM, pH 12, 85 °C 1:15 min, diluted 1:20), and purified, thermally treated uAgNPs (pH 12, 85 °C 1:15 min, diluted 1:20), all in MilliQ water. Absorption measurements were performed using a double-beam UV–Vis spectrophotometer (Lambda 650, PerkinElmer, Waltham, MA, USA). MilliQ water in a quartz cuvette was used as the reference. Absorption spectra were recorded over a wavelength range of 200–800 nm.

Fluorescent emission measurements were performed using a spectrofluorometer (FP-8300, JASCO, Tokyo, Japan). Emission spectra were acquired with excitation fixed at 260 nm and emission collected from 300 to 800 nm at 1 nm intervals. The excitation and emission bandwidths were both set to 2.5 nm, with a response time of 0.1 s, medium sensitivity, and scan speed of 500 nm·min^−1^. MilliQ water was used as the blank for baseline correction. Three-dimensional excitation–emission matrix (EEM) measurements were recorded under strictly matched preparation and instrumental conditions to discriminate between intrinsic protein fluorescence and emission arising from embedded silver species. Excitation wavelengths were scanned from 280 to 450 nm, and emission spectra were collected from 280 to 750 nm with a 1 nm interval. The excitation and emission bandwidths were set to 2.5 and 5 nm, respectively. Measurements were acquired at a scan speed of 1000 nm·min^−1^ with a 10 ms response time, medium sensitivity, and autogain enabled.

Fluorescence lifetime measurements were conducted using an Abberior (Göttingen, Germany) Expert Line fluorescence lifetime imaging microscopy (FLIM) system. BSA hydrogel (50 mg·mL^−1^, pH 12, 85 °C 1:15 min, diluted 1:20) and uAgNP–BSA hydrogel (pH 12, 85 °C 1:15 min, diluted 1:20) samples were prepared by homogenizing the material in water and mounting the suspension onto standard microscopy slides. Excitation was performed at 450 nm, and fluorescence emission was collected within the 533–558 nm spectral window. Fluorescence decay traces were acquired for 15 s and analyzed using SymPhoTime 64 (PicoQuant; Berlin, Germany) software. Fluorescence decay curves were fitted using a biexponential reconvolution model, selected as the optimal fit for all samples, and the average weighted intensity lifetime was obtained.

## Figures and Tables

**Table 1:** DLS results for AgNPs in water: Average volume-weighted size and PdI.

Sample	Peak 1 Diameter/nm ^1^	% Volume	Peak 2 Diameter/nm ^1^	% Volume	PdI
AgNPs 1:15	9.6 ± 6.5	97.4	42.4 ± 9.9	2.6	0.49
AgNPs 1:30	14.6 ± 4.4	92.1	50.7 ± 20.7	7.4	0.55
AgNPs 1:45	14.3 ± 4.9	93.7	49.0 ± 14.0	5.3	0.61
AgNPs/BSA liquid phase ^2^	18.8 ± 3.4	53.7	32.8 ± 8.0	41.4	0.64

^1^ Average hydrodynamic diameter (nm) ± SD from three independent measurements. ^2^ AgNPs were synthesized following the protocol described in [22].

## Data Availability

The data supporting the findings of this study are available at https://doi.org/10.5281/zenodo.18412845.

## Supplementary Materials

The following supporting information can be downloaded at https://www.mdpi.com/article/10.3390/gels12030231/s1: Figure S1: TEM and HR-TEM images. Figure S2: Circularity analysis of uAgNPs. Figure S3: EDX spectrum of the BSA hydrogel containing AgNPs. Figure S4: FTIR spectra of the BSA gels and uAgNP–BSA hydrogel composites. Figure S5: Absorption and emission spectra of the BSA gels and uAgNP–BSA hydrogel composites. Figure S6: Fluorescence emission decay traces of BSA hydrogel and BSA hydrogel containing uAgNPs.

## Abbreviations

The following abbreviations are used in this manuscript:

AgNPs	Silver nanoparticles
AuNPs	Gold nanoparticles
BSA	Bovine Serum Albumin
DLS	Dynamic Light Scattering
ECM	Extracellular Matrix
EDX	Energy-Dispersive X-ray Spectroscopy
EEM	Excitation–Emission Matrix
FCC	Face-Centered Cubic
FFT	Fast Fourier Transform
FLIM	Fluorescence Lifetime Imaging Microscopy
Fmoc-Phe	Fluorenylmethyloxycarbonyl-L-phenylalanine
FTIR	Fourier Transform Infrared spectroscopy
Phe–Phe	Diphenlyalanine
G′	Storage modulus
G″	Loss modulus
HRTEM	High-Resolution Transmission Electron Microscopy
LSPR	Localized Surface Plasmon Resonance
LVR	Linear Viscoelastic Regime
PdI	Polydispersity Index
PVP	Polyvinylpyrrolidone
ROI	Region of Interest
SPR	Surface Plasmon Resonance
TCSPC	Time correlated single photon counting
TEM	Transmission Electron Microscopy
uAgNPs	Ultrasmall silver nanoparticles
UV–Vis	Ultraviolet–Visible spectroscopy
