# Supplementary material for: Protein-Directed Nucleation and Stabilization of Ultrasmall Silver Nanoparticles Within BSA Hydrogels
Source: Gels. 2026 Mar 12;12(3):231. doi: 10.3390/gels12030231 (PMC13025548; doi:10.3390/gels12030231)
Supplement: Supplementary file 1 [file gels-12-00231-s001.zip › gels-4152887-supplementary.pdf]

SUPPLEMENTARY MATERIALS

Protein-Directed Nucleation and Stabilization of Ultrasmall Silver Nanoparticles within BSA Hydrogels

Carmen Salto-Giron [1], M. Carmen Gonzalez-Garcia [1], Mari C. Mañas-Torres [2,3], Modesto T. Lopez-Lopez [4,5], Luis Alvarez de Cienfuegos [2,5], Jose L. Hueso [6,7,8,9,10], Angel Orte [1,*] and Emilio Garcia-Fernandez [1,*]

1 Nanoscopy-UGR Laboratory, Departamento de Fisicoquímica, Unidad de Excelencia de Química Aplicada a Biomedicina y Medioambiente, Facultad de Farmacia, University of Granada, C. U. Cartuja, 18071 Granada, Spain; carmensalto@ugr.es (C.S.-G.); carmen.ggarcia@imdea.org (M.C.G.-G.)
2 Departamento de Química Orgánica, University of Granada, C. U. Fuentenueva, Avda. Severo Ochoa s/n, 18071 Granada, Spain; mariadelcarmen.manas@polymat.eu (M.C.M.-T.); lac@ugr.es (L.A.d.C.)
3 Current address: Basque Center for Macromolecular Design and Engineering (POLYMAT), Department of Applied Chemistry, Faculty of Chemistry, University of the Basque Country UPV/EHU, Paseo Manuel Lardizabal 3, 20018 Donostia-San Sebastián, Spain
4 Departamento de Física Aplicada, Research Unit "Modeling Nature" (MNat), University of Granada, C. U. Fuentenueva, 18071 Granada, Spain; modesto@ugr.es
5 Instituto de Investigación Biosanitaria Ibs.GRANADA, Avda. de Madrid 15, 18014 Granada, Spain
6 Instituto de Nanociencia y Materiales de Aragon (INMA), CSIC-Universidad de Zaragoza, Campus Rio Ebro, Edificio I+D, C/Poeta Mariano Esquillor, s/n, 50018 Zaragoza, Spain; jlhueso@unizar.es
7 Department of Chemical and Environmental Engineering, University of Zaragoza, C/María de Luna, 3, 50018 Zaragoza, Spain
8 Networking Research Center on Bioengineering, Biomaterials and Nanomedicine (CIBER-BBN), 28029 Madrid, Spain
9 Instituto de Investigación Sanitaria (IIS) de Aragón, Avenida San Juan Bosco, 13, 50009 Zaragoza, Spain
10 Escuela Politécnica Superior, University of Zaragoza, Crta. de Cuarte s/n, 22071 Huesca, Spain

* Correspondence: angelort@ugr.es (A.O.); emiliogf@ugr.es (E.G.F.)

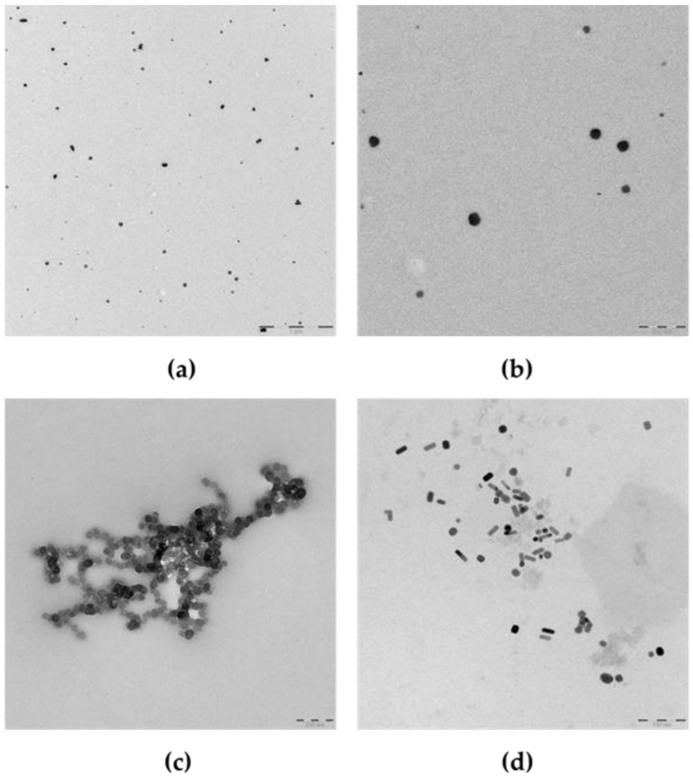

Figure S1. (a-c) Representative TEM images of uAgNP-BSA hydrogel composites obtained at different reaction times: 1 min 30 s (a-b), with (a) 1 µm and (b) 200 nm scale bars; and (c) 1 min 45 s heating time with 200 nm scale bar. (d) Representative TEM image of AgNPs prepared with BSA in liquid phase following the protocol in [1]. Scale bar represents 100 nm.

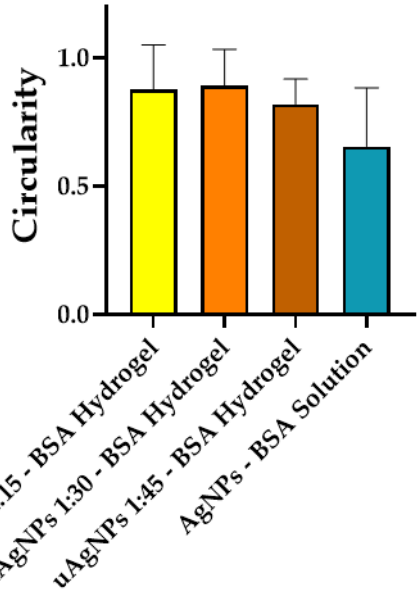

Figure S2. Mean circularity obtained in the particle analysis of TEM images of AgNPs in uAgNP-BSA hydrogel composites from different reaction times, and AgNPs prepared with BSA in liquid phase following the protocol in [1]. Circularity values ≥ 0.85 indicate high roundness, consistent with isotropic AgNP formation. Error bars represent SD from three independent preparations.

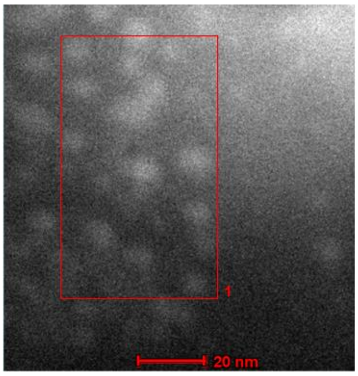

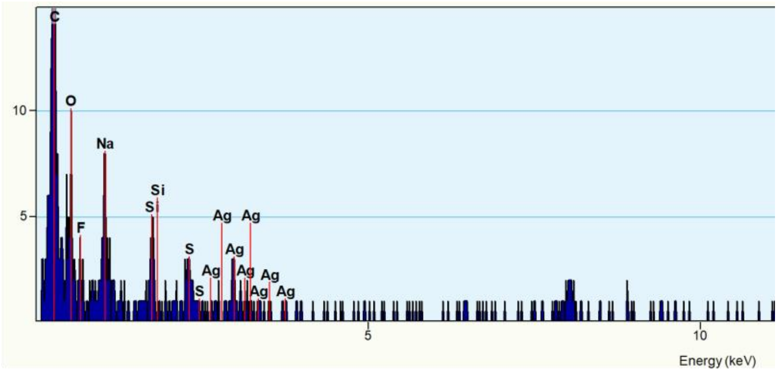

(a) (b)

Figure S3. EDX spectrum of the BSA hydrogel containing uAgNPs. Characteristic Ag peaks (Ag Lα, Lβ, Lγ, and M series) confirm the presence of metallic silver within the hydrogel. Peaks corresponding to C, O, S, and N arise from the BSA matrix, while Si, Na, and F originate from the TEM support grid and minor environmental contributions. The combination of EDX and HR-TEM data confirms the successful incorporation of crystalline uAgNPs within the BSA hydrogel network.

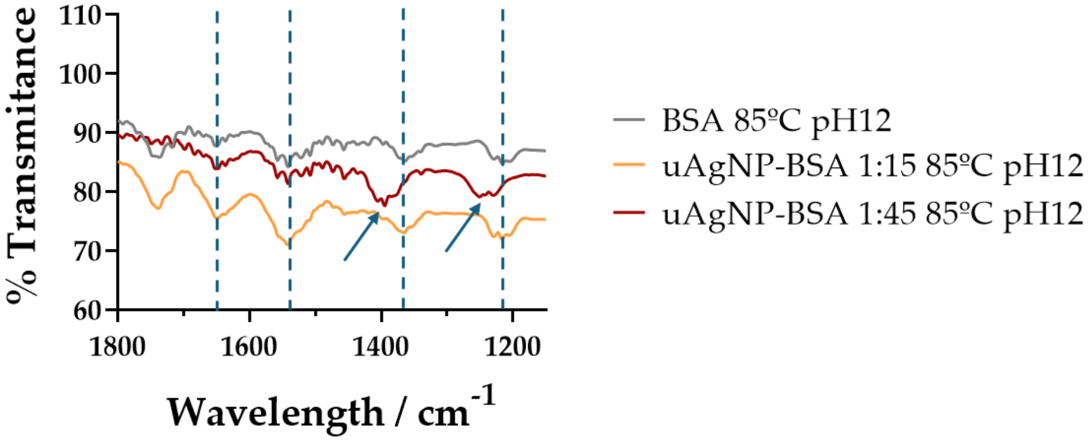

Figure S4. FTIR spectra of BSA hydrogel (gray) and the uAgNP–BSA hydrogel composites (1:15 min in orange and 1:45 min in red). Blue dashed lines mark the characteristic vibrational bands observed in the samples, while blue arrows indicate the band shifts specific to the uAgNP 1:45–BSA hydrogel relative to the reference BSA spectrum.

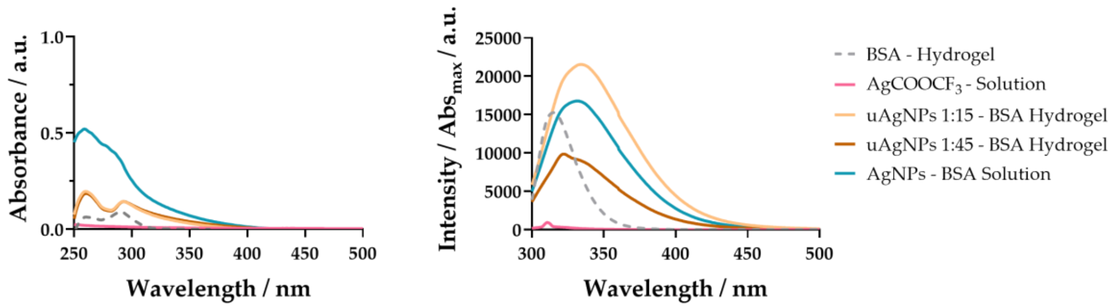

(a) **(b)**

Figure S5. Absorption (a) and emission relative to the maximum absorbance (b) spectra of BSA hydrogel 50 mg/mL (dash gray), AgCOOCF3 10 mM water solution (pink), uAgNPs 1:15–BSA hydrogel (light orange), uAgNPs 1:45–BSA hydrogel (brown), and AgNPs prepared in aqueous BSA (blue-green) [1] (b) Excitation wavelength, λ_{ex} = 260 nm.

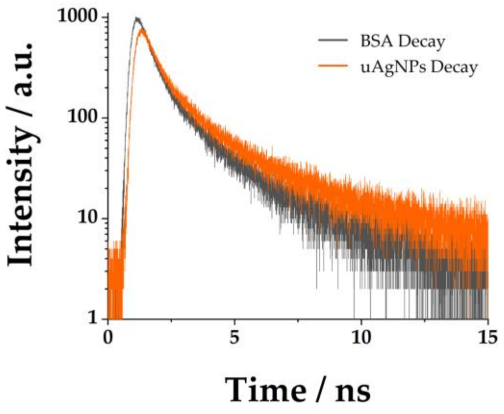

Figure S6. Time-resolved fluorescence decay profiles of BSA hydrogel 50 mg/mL (black) and uAgNPs 1:15–BSA hydrogel (orange). Excitation wavelength, λ_{ex} = 450 nm.

References

1. Basu, N.; Mandal, D. Time-dependent emission stokes shift in Au, Ag and Au/Ag fluorescent nanoclusters: evidence of multiple emissive states. *Photochemical & Photobiological Sciences* **2019**, *18*, 1782-1792, doi:10.1039/C8PP00540K.
